# Long-term myocardial recovery after mitral valve replacement in noncompaction cardiomyopathy

**DOI:** 10.1186/1749-8090-6-124

**Published:** 2011-09-30

**Authors:** Tariq Bhat, Thomas Costantino, Hilal Bhat, Yefim Olkovsky, Muhammad Akhtar, Sumaya Teli, Alfred Culliford

**Affiliations:** 1 Department of Medicine, Staten Island University Hospital, New York. 475 Seaview Ave, Staten Island New York 10305, USA; 2 Division of Cardiology, Staten Island University Hospital, New York. 475 Seaview Ave, Staten Island New York 10305, USA; 3 Department of Medicine, SKIMS, Soura, Kashmir 190011, India; 4 The Medical School, University of Sheffield, Beech Hill Road Sheffield, S10 2RX, UK; 5 Department of Cardiothoracic Surgery, NYU School of Medicine and Medical Center. 530 First Avenue, New York, NY 10016, USA

## Abstract

Isolated noncompaction of the left ventricle is a congenital cardiomyopathy, which has been described recently, with literature limited to case reports and case series. Even though various complications have been reported with noncompaction cardiomyopathy, among them severe mitral regurgitation has been reported recently in a few cases. There is no great evidence in the literature about its management, apart from some cases of mitral valve repair and replacement in young patients. We are reporting a case of an elderly lady with isolated left ventricular noncompaction cardiomyopathy associated with severe mitral regurgitation treated with mitral valve replacement with one and half year of follow up demonstrating significant myocardial recovery.

## Background

Isolated left ventricle noncompaction cardiomyopathy (ILVNC) is a rare congenital cardiomyopathy [1]. Severe mitral regurgitation has been reported recently in ILVNC [2]. There is no great evidence in the literature about its management. We are reporting a case of an elderly lady with ILVNC associated with severe mitral regurgitation treated with mitral valve replacement with one and half year (18 Months) of follow up, demonstrating significant improvement.

## Case Presentation

A 78-year-old lady presented with worsening heart failure (HF) symptoms. She had multiple prior hospitalizations for similar complaints. She had a history of atrial fibrillation, which was found in 1986 when she presented with embolic stroke and was also diagnosed with hypertrophic cardiomyopathy on echocardiogram. We believe this finding should have been diagnosed as ILVNC, but there was limited knowledge of this disorder at that time. Workup in the past for ischemic cardiomyopathy, including coronary angiogram, had been negative, however, now the patient had progressed to NYHA class IV HF. Two-dimensional and Doppler echocardiography (TTE) revealed decreased LV systolic function {ejection fraction (EF) = 30%} moderate to severe mitral valve regurgitation with a predominately posterior-directed jet. There was suspicion of ILVNC based on previous left ventriculogram. A transesophageal echocardiography (TEE) was done, which showed apical and posterior trabeculations, which met the criteria for ILVNC. Left and right cardiac catheterization and left ventriculography showed normal coronary arteries, severe pulmonary hypertension and extensive trabeculations consistent with ILVNC and severe mitral regurgitation. She was referred for mitral valve surgery.

Surgery was done through a median sternotomy. During surgery, repair of the mitral valve was not considered because of papillary muscle involvement. To preserve as many chordae tendenae as possible only portions of anterior and posterior leaflets were excised and replaced with St. Jude's biological tissue heart control device. She was discharged in a stable condition and noticed improvement in her symptoms. On a follow up visit at three months, the patient's symptoms had improved from NYHA class IV to NYHA class III, but 2 weeks after this visit she was admitted to the hospital for worsening heart failure symptoms and worsening left ventricular functioning with ejection fraction of (EF = 25%). The patient was managed with IV diuretics and was discharged home in stable condition. Repeat echocardiography 6 weeks later showed improvement in her left ventricular function. During subsequent follow-ups she has shown progressive improvement in both clinical and echocardiographic parameters. At one year of clinical and echocardiographic follow up after her mitral valve replacement, she showed a sustained and continuous improvement in her symptoms with no more hospitalizations for HF. After 18 months post valve surgery she remains NYHA class II with echocardiogram revealing left ventricular ejection fraction maintained at 45% with only trace mitral regurgitation.

## Discussion

Isolated noncompaction of the left ventricle a congenital cardiomyopathy, which is characterized by hypertrabeculations and deep recesses in the ventricular wall led by a defect in morphogenesis during embryogenesis [1]. ILVNC is a familial disorder but sporadic cases have also been reported [3]. Awareness about ILVNC has increased tremendously in the recent past more pertinently in the elderly population. In the absence of large studies and longer follow up, clinical features and long-term behavior of this disorder is ambiguous. Clinical presentation is variable and can be any combination of heart failure, arrhythmias, embolic events and conduction disorders [1]. Severe mitral regurgitation associated with ILVNC has been also been documented recently [2,4,5]. Long-term outcome of patients with ILVNC is not clear, but a recent small study showed worse prognosis than in the general population, but similar to dilated cardiomyopathy patients [6]. Due to an absence of sufficient evidence, diagnosis and treatment is still controversial, but echocardiography has been considered standard for diagnosis of noncompaction cardiomyopathy [1]. Jenni et al. [7] established four echocardiographic criteria for ILVNC diagnosis and all four are required for diagnosis. Other imaging modalities that can be diagnostic as well as determine the severity and prognosis are CMR, CCT and left ventriculography. Early diagnosis of ILVNC is important not only because of its high mortality in symptomatic patients, but also for screening relatives, as familial occurrence is known.

Management of patients with ILVNC is same as that of other cardiomyopathies that require treatment for heart failure, and appropriate prevention and management of complications that include arrhythmias, conduction abnormalities, systemic emboli and valvular dysfunction like severe mitral valve regurgitation [3]. There have been few cases reported of ILVNC associated with severe mitral regurgitation [2,4,5]. But due to limited data, appropriate management and their long-term outcome is not clear. There are reports of mitral reconstruction and replacement in young patients of ILVNC with some clinical improvements over a short term of follow up [4,5].

## Conclusion

This case report is first reported case of mitral valve replacement in elderly patient of ILVNC with one-year follow up showing a sustained improvement.

## Consent

Written informed consent was obtained from the patient for publication of this Case report and any accompanying images. A copy of the written consent is available for review by the Editor-in-Chief of this journal.

## Competing interests

The authors declare that they have no competing interests.

## Authors' contributions

AC, YO and TC analyzed and interpreted the patient data. TB, HB and ST were involved in doing the literature review and manuscript preparation and MA was also instrumental in obtaining informed consent. All authors have read and approved the final manuscript.
